# Maternal Stress and Effects of Prenatal Air Pollution on Offspring Mental Health Outcomes in Mice

**DOI:** 10.1289/ehp.1306560

**Published:** 2013-07-03

**Authors:** Jessica L. Bolton, Nicole C. Huff, Susan H. Smith, S. Nicholas Mason, W. Michael Foster, Richard L. Auten, Staci D. Bilbo

**Affiliations:** 1 Department of Psychology and Neuroscience, Duke University, Durham, North Carolina, USA; 2 Department of Pediatrics, Division of Neonatal Medicine, and; 3 Department of Medicine, Division of Pulmonary and Critical Care Medicine, Duke University Medical Center, Durham, North Carolina, USA

## Abstract

Background: Low socioeconomic status is consistently associated with reduced physical and mental health, but the mechanisms remain unclear. Increased levels of urban air pollutants interacting with parental stress have been proposed to explain health disparities in respiratory disease, but the impact of such interactions on mental health is unknown.

Objectives: We aimed to determine whether prenatal air pollution exposure and stress during pregnancy act synergistically on offspring to induce a neuroinflammatory response and subsequent neurocognitive disorders in adulthood.

Methods: Mouse dams were intermittently exposed via oropharyngeal aspiration to diesel exhaust particles (DEP; 50 μg × 6 doses) or vehicle throughout gestation. This exposure was combined with standard housing or nest material restriction (NR; a novel model of maternal stress) during the last third of gestation.

Results: Adult (postnatal day 60) offspring of dams that experienced both stressors (DEP and NR) displayed increased anxiety, but only male offspring of this group had impaired cognition. Furthermore, maternal DEP exposure increased proinflammatory interleukin (IL)-1β levels within the brains of adult males but not females, and maternal DEP and NR both decreased anti-inflammatory IL-10 in male, but not female, brains. Similarly, only DEP/NR males showed increased expression of the innate immune recognition gene toll-like receptor 4 (*Tlr4*) and its downstream effector, caspase-1.

Conclusions: These results show that maternal stress during late gestation increases the susceptibility of offspring—particularly males—to the deleterious effects of prenatal air pollutant exposure, which may be due to a synergism of these factors acting on innate immune recognition genes and downstream neuroinflammatory cascades within the developing brain.

Citation: Bolton JL, Huff NC, Smith SH, Mason SN, Foster WM, Auten RL, Bilbo SD. 2013. Maternal stress and effects of prenatal air pollution on offspring mental health outcomes in mice. Environ Health Perspect 121:1075–1082; http://dx.doi.org/10.1289/ehp.1306560

## Introduction

Although low socioeconomic status (SES) has been repeatedly associated with a higher rate of chronic health problems and mental disorders (Adler and Rehkopf 2008; Reijneveld and Schene 1998), explicit characterization of the factors that underlie this phenomenon remains elusive (Evans and Kantrowitz 2002). A growing body of research suggests that maternal well-being during pregnancy is a crucial determinant of lifelong physical and mental health of the offspring (Case et al. 2005; Hackman et al. 2010; Susser et al. 1999). Notably, expectant mothers living in low-SES conditions experience the greatest burden of toxicants (Evans and Kantrowitz 2002), along with fewer resources and high psychological stress (Seguin et al. 1995). Exposures to toxicants such as lead are well known to adversely affect brain development (Needleman et al. 1990; Weiss and Landrigan 2000). However, “social toxins,” such as violence, poverty, and other factors that generate psychological stress in low-SES parents and children, have only recently begun to gain recognition as risk factors that can also alter the trajectory of brain development (Wright 2009). For example, Clougherty et al. (2007) noted an association between air pollution and asthma only in children who were also living with a chronic stressor (e.g., domestic violence). Similarly, parental stress can increase the effect of *in utero* exposures to toxicants (i.e., tobacco smoke) on childhood asthma risk (Shankardass et al. 2009). In such cases, stress may increase vulnerability, permitting a toxicant to initiate significant injury to physiological systems greater than any injury resulting from exposure to the toxicant alone. Importantly, these synergistic effects of stress and pollutants are possible because they likely act on common biological systems, such as innate immune pathways (Frank et al. 2007; Levesque et al. 2011) within the developing nervous system.

Air pollution, one of the most relevant and pervasive environmental toxicants in the modern world, is a particularly important threat to child health and is increasingly associated with neurodevelopmental disorders such as autism (Volk et al. 2013). Mechanistic studies have revealed that diesel exhaust, a major component of air pollution, markedly activates microglia, the resident immune cells of the brain, in adult rats (Levesque et al. 2011). Furthermore, we observed in mice that maternal exposure to diesel exhaust causes long-term increases in microglial antigen expression in the brains of adult offspring (Bolton et al. 2012). Notably, prior stress has been shown to enhance proinflammatory cytokine expression and associated neural damage following an immune challenge in adult rats (De Pablos et al. 2006), most likely by sensitizing microglia (Frank et al. 2011). Because cytokines are important for normal brain development and adult function (Boulanger 2009; Yirmiya and Goshen 2011), significant perturbations in their expression may have enduring consequences for lifelong mental health (Dantzer et al. 2008).

We hypothesized that the addition of maternal stress to the impact of prenatal air pollution exposure would act synergistically in offspring to impair mental health outcomes, compared with the effects of either exposure alone. To test this hypothesis, we combined our animal model of prenatal diesel exhaust exposure (Auten et al. 2012) with an adaptation of a novel model of maternal resource deprivation [nest restriction (NR)] (Rice et al. 2008). Pregnant mice exposed by intermittent aspiration to vehicle or diesel exhaust particles (DEP) throughout gestation either lived in normal housing or had reduced nesting materials during the last one-third of pregnancy.

## Methods

*Animals*. We obtained adult male and female C57BL/6 mice from Charles River Laboratories (Raleigh, NC) and time-mated them in four separate cohorts (see Supplemental Material, p.2). After confirming successful mating of animals [vaginal plug, considered to be embryonic day (E) 0], we paired females in individually ventilated cages with specialized bedding (AlphaDri; Shepherd Specialty Papers, Milford, NJ) and *ad libitum* access to food (PicoLab Mouse Diet 5058; Lab-Diet, Philadelphia, PA) and filtered water. Each of the four cohorts was used for a separate analysis: *a*) cytokine protein in fetal brain, *b*) gene expression in postnatal day (PND) 30 brain, *c*) adult behavior and brain cytokine protein, and *d*) adult microglial isolations and gene expression. In addition, one pup was randomly selected at PND1 from litters across the second and third cohorts for corticosterone (CORT) analysis. All mice used in this study were treated humanely and with regard for alleviation of suffering, and experiments were conducted using protocols approved by the Duke University Animal Care and Use Committee.

*Prenatal stressors.* DEP exposures. Beginning on E2, time-mated females were lightly anesthetized with 2% isoflurane for approximately 1 min and treated with DEP via oropharyngeal aspiration (Auten et al. 2012) (see Supplemental Material, pp. 2–3). Females received 50 μg DEP suspended in 50 μL vehicle [phosphate buffered saline (PBS), pH 7.2, 0.05% Tween-20] or vehicle alone (VEH) every 3 days during E2–E17 for a total of six doses, as a model of intermittent exposure. This dose and route of delivery induces maternal lung inflammation (e.g., white blood cell infiltration) comparable to levels observed following intermittent maternal inhalation of diesel exhaust at environmentally relevant concentrations (Auten et al. 2012). Moreover, both routes of delivery result in similar levels of particle deposition within the lung (Foster et al. 2001).

Maternal resource deprivation. We adapted a model of postnatal NR by applying the method of Rice et al. (2008) to the prenatal period, a degree of restriction that produced minimal behavioral changes with NR alone. On E14, after DEP exposures, half of the VEH- and DEP-treated dams were singly housed in clean cages with a thin layer of bedding under an elevated fine-gauge aluminum mesh platform (mesh dimensions, 0.4 cm × 0.9 cm; McNichols Co., Tampa, FL) and provided with two-thirds of one square of felt-like nesting material (~ 1.9 g; NR group). The remaining dams were singly housed in clean cages with bedding and one full square of nesting material (~ 2.8 g; control group). On E19, we placed NR dams in clean cages with normal bedding and one full square of nesting material; from that point on, NR animals were treated identically to control dams. This design resulted in four groups of dams: VEH/control (*n* = 8), DEP/control (*n* = 10), VEH/NR (*n* = 8), and DEP/NR (*n* = 10).

*Neonatal outcomes and maternal behavior.* Birth weights. All animals were allowed to deliver spontaneously on E19–E20 (defined as PND0), and offspring were not cross-fostered. To acquire a litter average for birth weight (*n* = 8–10 litters/group from three cohorts), we weighed pups (sex not determined) on PND1. Four weeks later, offspring were weaned into clean cages of two to five same-sex siblings and provided with *ad libitum* access to standard chow and filtered water.

Neonatal CORT measurement. On PND1, we randomly selected one pup from each litter (males, *n* = 5–8/group; females, *n* = 3–7/group from two cohorts), performed rapid decapitation, and collected trunk blood to obtain a measure of basal circulating CORT levels soon after birth. We assessed total serum CORT concentrations using an enzyme-linked immunosorbent assay (ELISA; Enzo Life Sciences Inc., Ann Arbor, MI) (Bilbo et al. 2007).

Maternal behavior assessment. To characterize the effect of prenatal stressors on maternal care, we observed dams (*n* = 3–7/group from two cohorts) twice daily with their litters on PND2–PND9 to determine time spent on the nest, nursing, and licking and grooming their pups (Myers et al. 1989) (see Supplemental Material, p. 4).

*Fetal brain cytokine analysis.* At E18, VEH- or DEP-treated dams with or without NR (*n* = 2–3/group from one cohort) were euthanized by sodium pentobarbital anesthesia [250 mg/kg by intraperitoneal injection (i.p.)]. Fetuses were removed by hysterotomy, placed on ice, and decapitated. Whole fetal brains were snap-frozen and stored at –80°C until processing. To determine the sex of each fetus, we extracted genomic DNA (Kouduka et al. 2006) from tail snips for later genotyping.

We measured interleukin (IL)-1β, a proinflammatory cytokine, and IL-10, an anti-inflammatory cytokine, in lipid-depleted fetal brain homogenates normalized to total protein (200 μg/well; *n* = 7–8 brains/sex/group) (see Supplemental Material, p. 3). We selected these cytokines because of their important role in microglial function, brain development, and behavior (Deverman and Patterson 2009; Williamson et al. 2011; Yirmiya and Goshen 2011).

*PND30 neuroimmune gene expression.* To determine the long-term effect of prenatal stressors on genes critical for innate immune recognition and the subsequent cytokine response in the brain, we assessed male (*n* = 8–13 pups from 2–4 litters per treatment group from one cohort) and female (*n* = 5 pups from 2–4 litters per treatment group from one cohort) offspring at PND30. Mice were deeply anesthetized with ketamine (430 mg/kg) and xylazine (65 mg/kg; i.p.) and transcardially perfused with ice-cold saline for 2 min to clear brains of blood. Afterward, we extracted the brains, removed the cerebellum and hindbrain, and cut the remaining forebrain sagittally in half. Half brains were snap frozen and stored at –80°C until analysis by quantitative real-time polymerase chain reaction (qRT-PCR) as described by Williamson et al. (2011) (see Supplemental Material, p. 4 and Table S1).

*Behavioral procedures.* We assessed behavioral outcomes as a result of prenatal stressors in young adult (PND60–PND90) male and female offspring (*n* = 7–9 animals/sex from 2–4 litters per treatment group from one cohort) using a sequence of behavioral tests, with 1 week between each test. We tested males and females separately and performed all testing during the dark cycle (between 1000 hours and 1600 hours). Throughout testing, we also monitored females’ estrous cycles.

Contextual and auditory cue fear conditioning. We assessed memory as described in detail for rats (Williamson et al. 2011), but with slight modifications for mice (see Supplemental Material, pp. 5–6). Briefly, mice were trained to associate a foot shock with a specific context and an auditory tone. We tested each mouse’s memory of these associations 48 hr later by assessing freezing behavior (the prototypical rodent fear response) in the fear context, in a new context, and in response to the auditory cue [conditioned stimulus (CS)] in the new context. We used contextual fear conditioning to assess hippocampal-dependent memory, which has been reported to be uniquely vulnerable to early-life insults (Williamson et al. 2011), compared with auditory cue fear conditioning, which does not require the hippocampus (Phillips and LeDoux 1992).

Elevated zero-maze. We assessed anxiety-like behavior using an adaptation of a widely used method for rodents that measures time spent in the closed versus open arms of a circular maze (Shepherd et al. 1994) (see Supplemental Material, p. 6). Immediately after the test (< 5 min), we collected a blood sample (~ 100 μL) from the facial vein of each mouse (in a separate room). We determined CORT levels in these blood samples by ELISA, as described above.

Forced swim test. We assessed depressive-like behavior of the mice by measuring time spent immobile in a container of water (Castagné et al. 2010) (see Supplemental Material, p. 6).

*Adult brain cytokine analysis.* To determine the enduring effects of prenatal stressors on brain cytokines and their potential role in observed behavioral changes, we assessed cytokine levels in the brains of adult offspring 10 days after behavioral testing. Offspring (*n* = 7–9/group/sex) were anesthetized and perfused as described above. We then extracted brains and dissected them on ice into hypothalamus (HYP), prefrontal cortex (PFC), hippocampus (HIPP), and adjacent parietal cortex (PCX). We selected these regions for their known roles in the cognitive and affective behaviors that we assessed. To obtain enough total protein for analysis, we pooled the dissected regions from each animal, snap-froze them, and stored them at –80°C until processing. We performed ELISA protein analyses of IL-1β and IL-10 as described above.

*Microglial isolation and gene expression analysis.*We determined the cellular source of the measured cytokines in brain tissue from adult (~ PND60) behaviorally naïve offspring (*n* = 5/sex from 2–3 litters/group from one cohort). We again pooled dissected HYP, PFC, HIPP, and PCX tissue from each animal to obtain enough cells for later analysis. We isolated microglia by magnetic-activated cell sorting (Williamson et al. 2011), using the Neural Tissue Dissociation Kit (P), anti-myelin microbeads, and anti-CD11b (an established marker for microglia) microbeads, all from Miltenyi Biotec Inc. (Auburn, CA). We then washed cells in sterile PBS and stored them at –80°C until analysis by qRT-PCR as described by Williamson et al. (2011) (see Supplemental Material, p. 6).

*Data analysis.* We analyzed all data using SPSS statistical software (IBM, Armonk, NY). Because of heterogenous variance, neonatal CORT data were log-transformed. For ELISA analyses, samples that had undetectable levels of IL-1β or IL-10 were assigned a value of one-half the lowest detectable value in the assay (Thompson et al. 2012). We used three-way analysis of variance (ANOVA) (sex × DEP × NR) to analyze all data, except for the PCR data from CD11b-positive (CD11b+) and CD11b-negative (CD11b–) isolated cells, for which we used four-way ANOVA (sex × DEP × NR × cell population). We followed up interactions with sex using separate 2-way ANOVA (DEP × NR) for males and females to identify sex-specific effects. We also followed up significant DEP × NR interactions within each sex using post hoc comparisons [Tukey’s HSD (honestly significant difference)] to identify group differences, assuming significance at *p <* 0.05. All reported *p-*values are two-tailed, except for the correlations between behavioral measures and cytokine measures from adult brains; for those we used one-tailed *p*-values because we had clear *a priori* hypotheses based on the apparent correspondence between observed group differences in the two measures. Finally, we controlled for litter effects by using multiple litters per treatment group. In addition, litter effects can be excluded because of the sex-specific effects observed in males and females from the same litter.

## Results

**Figure 1:**
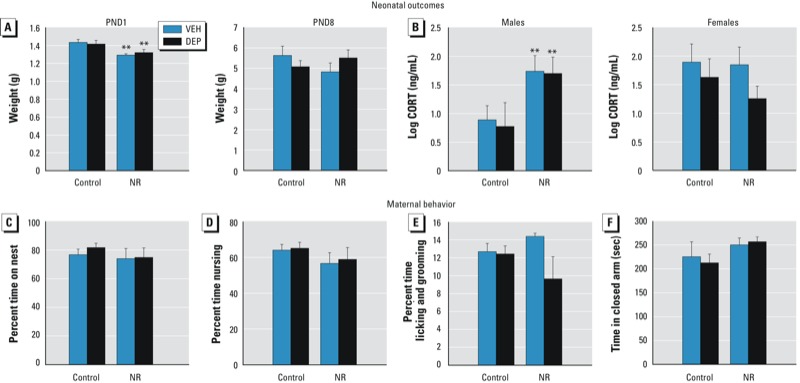
Effects of prenatal DEP and nest restriction (NR) on neonatal outcomes (*A,B*) and maternal behavior (*C–F*). (*A*) Prenatal NR decreased body weights in PND1 pups, but weights normalized by PND8. (*B*) Prenatal NR increased serum CORT in PND1 male but not female pups. (*C–E*) Neither DEP nor NR altered the percent of time dams spent on the nest (*C*), nursing (*D*), or licking and grooming (*E*) their pups during PND2–PND9. (*F*) DEP and NR had no enduring effects on maternal anxiety-like behavior 60 days postpartum. Data are mean ±  SE of 8–10 mice/group for pup weights, 3–8 mice/group for PND1 CORT, and of 3–7 mice/group for maternal behavior. **p < 0.05 compared with control groups.

*Neonatal outcomes and maternal behavior.* Previous research has shown that postnatal NR results in more frequent dam departures from the nest, decreased pup weights, and increased plasma CORT at PND9 (Rice et al. 2008). In our adaptation of the model, prenatal NR decreased birth weights [main effect of NR, *F*(1,32) = 12.09, *p* < 0.005] regardless of prenatal DEP exposure, but weights normalized by PND8 (Figure 1A). Importantly, we observed no significant differences in litter size or composition due to either environmental stressor (see Supplemental Material, Table S2). In addition to the effect on birth weight, prenatal NR also increased PND1 serum CORT in male pups [main effect of NR, *F*(1,20) = 7.89, *p <* 0.05] but not in females [sex × NR interaction, *F*(1,35) = 2.78, *p* < 0.05] (Figure 1B). However, unlike postnatal NR, prenatal NR did not affect maternal behavior during PND2–PND9, a critical period for changes in maternal care to affect offspring brain development (Avishai-Eliner et al. 2001). Specifically, we observed no significant group differences in the percentage of time dams spent on the nest (Figure 1C) or in nursing (Figure 1D) or licking and grooming their pups (Figure 1E). Furthermore, prenatal stressors did not have any enduring effects on maternal anxiety-like behavior when dams were tested in the elevated zero-maze 60 days postpartum (Figure 1F).

**Figure 2:**
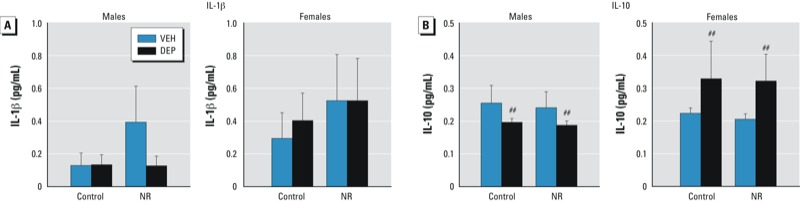
Effects of prenatal DEP and NR on the cytokine response in fetal brain. (*A*) No significant differences in IL‑1β protein levels were detected between treatment groups in the E18 brain. (*B*) A sexually dimorphic IL‑10 response was observed in the brains of fetuses prenatally exposed to DEP: Male brains exhibited a down-regulation of IL‑10, whereas female brains exhibited up-regulation. Data presented are mean ± SE of 7–8 animals/group. ^##^*p* = 0.1 compared with the corresponding VEH group; for sex × DEP interaction, *p* < 0.05.

*Fetal brain cytokine analysis.* The proinflammatory cytokine IL-1β was not detectable in most of the E18 brain samples, and we observed no significant group differences due to sex or prenatal stressors (Figure 2A). In contrast, the anti-inflammatory cytokine IL-10 was detectable in a greater proportion of samples, and there was a significant sex × DEP interaction [*F*(1,55) = 4.24, *p* < 0.05]. Follow-up tests revealed that males tended to down-regulate IL-10 in response to DEP exposure [trend for main effect of DEP, *F*(1,27) = 2.43, *p* = 0.1], whereas females tended to up-regulate IL-10 in response to DEP [trend for main effect of DEP, *F*(1,28) = 2.395, *p* = 0.1] (Figure 2B).

**Figure 3:**
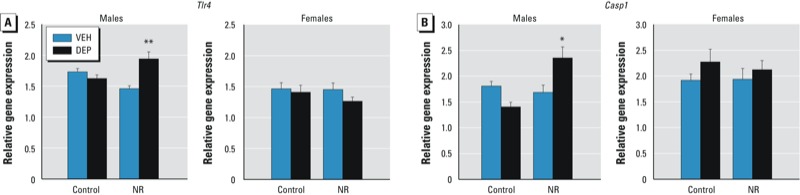
Effects of prenatal DEP and NR on neuroimmune gene expression at PND30. The brains of DEP/NR males displayed increased *Tlr4* (*A*) and *Casp1* (*B*) expression, whereas DEP/NR female brains did not. Data presented are mean ± SE of 5–13 animals/group. *p < 0.05 vs. all other groups. **p < 0.05 compared with DEP/control and VEH/NR.

*PND30 neuroimmune gene expression.* A preliminary mouse inflammatory response 84-gene PCR array performed on PND30 male brains, identified *Tlr4* as the only gene that exhibited a synergistic effect of DEP and NR (data not shown). Notably, TLR4 is an innate immune receptor critical for the response to both environmental toxicants and stress (Arbour et al. 2000; Caso et al. 2008). We replicated this result with single-analyte qRT-PCR, finding a significant DEP × NR interaction [*F*(1,34) = 16.79, *p* < 0.001] for PND30 male brains. Specifically, the DEP/NR group had significantly higher *Tlr4* expression than did the DEP/control and VEH/NR groups (*p* < 0.05; Figure 3A). Interestingly, we observed no significant differences among PND30 females [sex × DEP × NR interaction, *F*(1,51) = 9.18, *p* < 0.005]. In addition, expression of caspase-1 (*Casp1*), a downstream effector molecule and key enzyme for IL-1β production (Black et al. 1988), exhibited a similar pattern to *Tlr4* expression. Post hoc tests revealed that DEP/NR male brains had significantly higher expression of *Casp1* than all other groups [DEP × NR interaction, *F*(1,37) = 14.66, *p* < 0.001; post hoc, *p* < 0.01], whereas there were no significant differences among females [sex × DEP × NR interaction, *F*(1,54) = 6.45, *p* < 0.05] (Figure 3B).

**Figure 4:**
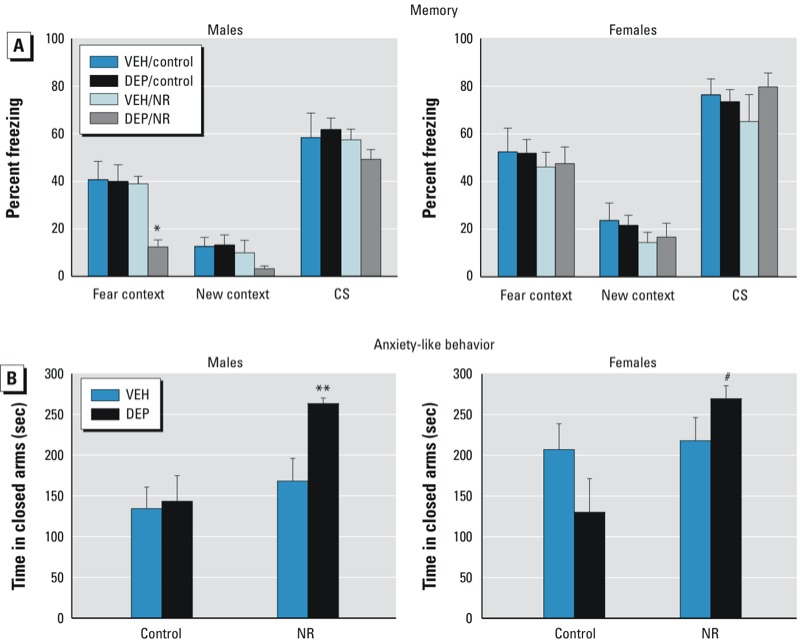
Effects of prenatal DEP and NR on cognitive and affective behavior of adult mice. (*A*) DEP/NR males displayed decreased freezing in the fear context, which indicates a hippocampal-dependent memory deficit, whereas DEP/NR females did not display this behavior. (*B*) Both male and female DEP/NR offspring spent more time in the closed arm, which indicates anxiety-like behavior. Data presented are mean ± SE of 7–9 mice/group. *p < 0.05 compared with all other groups. **p < 0.05 compared with DEP/control and VEH/control; *p* = 0.07 compared with VEH/NR. ^#^*p* < 0.05 compared with DEP/control).

*Memory.* When assessed for contextual fear recall, DEP/NR males froze significantly less than did all other groups in the fear context [DEP × NR interaction, *F*(1,29) = 5.15, *p* < 0.05; post hoc, *p* < 0.05]. However, DEP/​NR females exhibited no such hippocampal-dependent memory impairment [trend for sex × DEP × NR interaction, *F*(1,57) = 2.23, *p* = 0.1] (Figure 4A). Importantly, this male-specific deficit was not due to generalized hyperactivity or an inability of the DEP/NR males to freeze, because there were no significant differences in freezing in a novel context (“new context”) or freezing to the auditory cue (CS) (Figure 4A). Furthermore, the females’ stage of the estrous cycle did not significantly affect their behavior in any of the tests (data not shown).

*Anxiety- and depressive-like behavior.* In the elevated zero-maze, both adult male and female DEP/NR offspring spent more time in the closed arms, indicative of increased anxiety [DEP × NR interaction with sexes combined, *F*(1,55) = 7.350, *p* < 0.01] (Figure 4B). Post hoc tests revealed that DEP/​NR males were significantly more anxious than were VEH/control and DEP/​control males (*p* < 0.05). In addition, there was a trend for DEP/NR males to be more anxious than VEH/NR males (*p* = 0.07), which did not significantly differ from VEH/control males. DEP/​NR females were more anxious than DEP/​control females (*p <* 0.05), whereas VEH/control and VEH/NR females did not differ from each other. However, this increase in anxiety in the DEP/​NR animals was not associated with increased serum CORT because NR males exhibited a slight decrease in CORT immediately after the test, regardless of prenatal DEP exposure [main effect of NR, *F*(1,20) = 5.286, *p* < 0.05], and females displayed no significant differences (see Supplemental Material, Figure S1A). Finally, neither males nor females displayed any significant group differences in the forced swim test (see Supplemental Material, Figure S1B).

**Figure 5:**
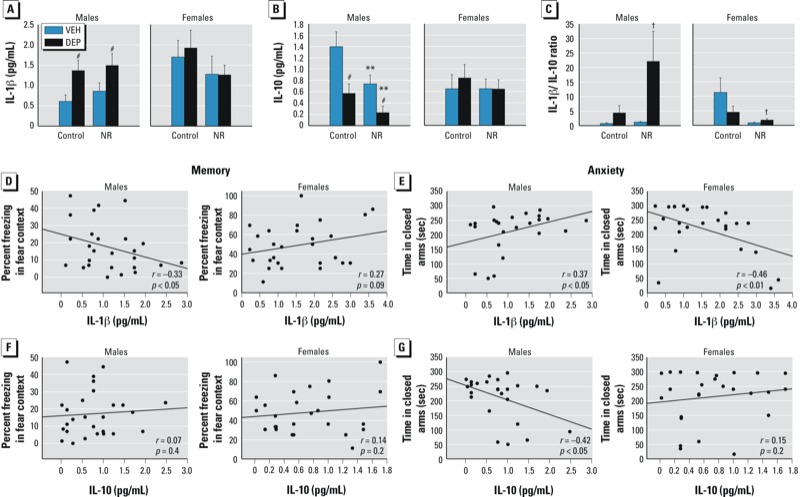
Effects of prenatal DEP and NR on cytokine levels in the adult brain. (*A*) DEP-exposed males exhibited a significant increase in brain IL‑1β, but DEP-exposed females did not. (*B*) Males exhibited a significant decrease in brain IL‑10 concentration in response to both DEP and NR, but no significant changes were observed in females. (*C*) Overall, DEP/NR males exhibited a greater proinflammatory bias (IL‑1β/IL‑10 ratio) than DEP/NR females. Data for *A–C* are presented as the mean ± SE of 5–8 animals/group. (*D–E*) Brain IL‑1β concentration was significantly correlated with memory (*D*) and anxiety (*E*) measures in both males and females, although in opposite directions. (*F*) Brain IL‑10 concentration was not correlated with memory performance in either males or females. (*G*) Brain IL‑10 concentration was negatively correlated with anxiety-like behavior in males but not in females. Data points in *D–G* represent correlated measures for individual animals from one entire cohort (*n* = 24–27 total animals per sex). ^#^*p* < 0.05 DEP compared with VEH groups. **p < 0.05 NR compared with both control groups. ^##^*p* = 0.08, DEP/NR males compared with DEP/NR females; significant sex × DEP and sex × NR interactions (*p* < 0.05).

*Adult brain cytokine analysis.* Ten days after behavioral testing, male brains from DEP groups exhibited increased levels of IL-1β protein [main effect of DEP, *F*(1,22) = 7.84, *p* < 0.01], whereas there were no group differences among female brains [trend for sex × NR interaction, *F*(1,45) = 2.49, *p* = 0.1] (Figure 5A). In contrast, maternal DEP exposure and NR resulted in decreased levels of IL-10 protein, in an additive fashion, in male brains [main effect of DEP, *F*(1,22) = 12.13, *p* < 0.005; main effect of NR, *F*(1,22) = 6.89, *p* < 0.05], whereas female brains again did not exhibit any significant group differences due to prenatal stressors [sex × DEP interaction, *F*(1,45) = 7.10, *p* < 0.05] (Figure 5B). Overall, DEP/NR males exhibited a greater proinflammatory bias (IL-1β/IL-10 ratio) (de Wit et al. 2010) than did DEP/NR females [sex × DEP interaction, *F*(1,39) = 4.26, *p* < 0.05; sex × NR interaction, *F*(1,39) = 4.53, *p* < 0.05; post hoc, *p* = 0.08] (Figure 5C).

We found it striking that the group differences in brain cytokine measures seemed to parallel the behavioral differences we had observed in the same animals, and indeed, further analyses revealed that the brain levels of IL-1β and IL-10 correlated significantly with the behavioral measures of memory and anxiety, although in a divergent manner in males and females. Males exhibited a significant negative correlation between IL-1β and contextual fear memory [*r*(26) = –0.33, *p* < 0.05], such that higher levels of proinflammatory IL-1β were associated with decreased freezing in the fear context, whereas females showed a trend for a positive correlation [*r*(27) = 0.27, *p* = 0.09] (Figure 5D). IL-1β was also positively correlated with anxiety-like behavior in males [*r*(24) = 0.37, *p* < 0.05], such that higher levels of IL-1β were associated with increased time spent in the closed arms of the elevated zero-maze, whereas in females IL-1β was negatively correlated with anxiety-like behavior [*r*(26) = –0.46, *p* < 0.01] (Figure 5E). On the other hand, anti-inflammatory IL-10 exhibited no significant correlations with memory in males or females (Figure 5F), but it was negatively correlated with anxiety-like behavior in males [*r*(25) = –0.42, *p* < 0.05] and not females (Figure 5G).

*Gene expression of isolated CD11b+ and CD11b– cells.* To assess the purity of the isolated cells, we confirmed that *Cd11b* mRNA was expressed only in CD11b+ cells, as expected [main effect of cell population, *F*(1,64) = 71.68, *p* < 0.001] (see Supplemental Material, Figure S2A). In contrast, CD11b– cells expressed approximately 20-fold higher levels of *Bdnf* (brain-derived neurotrophic factor) mRNA and *Gfap* (glial fibrillary acidic protein) mRNA than did CD11b+ cells (all *p*-values < 0.001; see Supplemental Material, Figure S2B and C, respectively), indicating that this cell population (~ 80% of total cells) contains astrocytes and neurons. Isolated CD11b+ cells (microglia) had approximately 170-fold higher levels of *Il1*β mRNA and approximately 80-fold higher levels of *Il10* mRNA than did CD11b– cells (all *p*-values < 0.001; see Supplemental Material, Figure S2D,E). In addition, CD11b+ cells expressed approximately 40-fold more *Casp1* and approximately 20-fold more *Tlr4* than CD11b– cells (all *p*-values < 0.001; see Supplemental Material, Figure S2G and F, respectively). However, we observed no significant group differences in gene expression due to sex or prenatal stressors.

## Discussion

We observed that maternal stress during late pregnancy exacerbated the impact of *in utero* exposure to DEP on mental health outcomes in adult offspring—outcomes that are associated with alterations in neuroinflammatory tone (i.e., increases in IL-1b and decreases in IL-10). The adult DEP/NR males (prenatally exposed to both stressors) exhibited significant memory deficits and anxiety, whereas DEP/​NR females displayed only slightly increased anxiety. Importantly, the impact of prenatal events on offspring behavior is always complex and may involve changes in several physiological pathways, as well as potential alterations in maternal–offspring interactions after birth—both of which can profoundly modify neural development (Caldji et al. 1998). In the present study, NR males (exposed to maternal stress) had reduced birth weight and increased CORT on PND1, independent of maternal DEP exposure. However, maternal care was not significantly altered by resource deprivation, and we observed no changes in maternal anxiety as a consequence of treatment. We observed no differences in corticosterone in adult offspring that could explain behavioral changes in response to the combined stressors. Instead, the alterations in brain cytokines, which occurred in a sexually dimorphic manner, may underlie distinct behavioral phenotypes in adult male and female offspring. An optimal balance of central proinflammatory cytokines, such as IL-1β, is critical for mental health, including mood regulation and hippocampal-dependent learning and memory (Yirmiya and Goshen 2011). In contrast, high levels of anti-inflammatory IL-10 are protective against behavioral changes due to microglial-driven neuroinflammation (Schwarz et al. 2011). Therefore, the diminished IL-10, in combination with increased IL-1β, that we observed in DEP/NR males could affect their relative vulnerability to cognitive impairments and mood dysregulation, compared with DEP/​NR females, which did not exhibit such a proinflammatory bias. The underlying mechanism of this sex difference warrants further exploration. Importantly, there is a male bias in the prevalence of neurodevelopmental disorders, including learning disabilities (Flannery et al. 2000) and autism (Stone et al. 2004), in addition to differences between the sexes in childhood outcomes following maternal stress during pregnancy (Cao et al. 2012; Fang et al. 2011).

Our data from isolated CD11b+ and CD11b– cells demonstrate that microglia—​not neurons or astrocytes—are the primary source of the measured cytokines in the brain, suggesting that they are a target of “programming” by the prenatal stressors. Microglia begin to colonize the rodent brain around E9–E10 (Ginhoux et al. 2010) and critically shape several aspects of normal brain development. Importantly, microglia largely remain in an activated, amoeboid state until the early postnatal period (Ling and Wong 2004), which makes them especially sensitive to long-term functional changes by perinatal inflammatory events (Bilbo and Schwarz 2009; Williamson et al. 2011). However, we did not detect any significant differences in cytokine gene expression in isolated adult microglia due to prenatal stressors, despite the clear differences in cytokine protein. This discrepancy may stem from the fact that the animals we used for protein analysis underwent behavioral testing, whereas the animals used for CD11b isolation and gene expression analysis were behaviorally naïve. It is possible that behavioral testing may serve as a sufficient stressor to elicit relatively long-term increases in cytokine levels (i.e., enduring until tissue collection) in the brains of the DEP/NR animals, which would not be observed at baseline. Alternatively, there may be additional regulatory mechanisms at work. For instance, the selective increase in caspase-1 expression in DEP/NR males supports a role for the inflammasome, which is critical for the cleavage of proIL-1β into its mature form (Latz 2010), although we did not examine this directly. Importantly, TLR4 signaling is required for the activation of the inflammasome (Bauernfeind et al. 2009). Furthermore, DEP and maternal stress-induced signals may converge on microglia via TLR4, which was predominantly expressed by microglia, consistent with our previous findings in rats (Schwarz and Bilbo 2013), and is exaggerated in the brains of DEP/NR males. TLR4 is an important innate immune receptor that recognizes pathogen-associated molecular patterns (e.g., lipopolysaccharide), as well as endogenous danger-associated molecular patterns released in response to cellular distress (e.g., DEP-induced hyaluronan or high-mobility group box 1) (Bianchi 2007). Notably, glucocorticoids may up-regulate TLRs on microglia, augmenting subsequent neuroinflammatory responses (Frank et al. 2010; Garate et al. 2013). Thus, *Tlr4* up-regulation may occur to a greater extent in males than in females as a result of the significant increase in serum corticosterone in males born to stressed dams.

Although the alterations in IL-1β we observed in adult offspring were not present in the fetal brain, we did observe a significant sex difference in IL-10 in the E18 brains of DEP-exposed pups. Specifically, although DEP males exhibited a down-regulation of IL-10, DEP females displayed an up-regulation of this anti-inflammatory cytokine, suggesting that IL-10 may have been protective against the neurodevelopmental effects of prenatal stressors. Along with brain cytokines, the placenta has been increasingly implicated as a key player in brain development, as well as in the fetal response to prenatal insults (Hsiao and Patterson 2012), and warrants further exploration in our model. Thus, our working hypothesis is that maternal stress-induced changes in TLR4 signaling enhance the effects of a chemical exposure such as DEP, likely involving the maternal–placental–fetal interface (Auten et al. 2009, 2012), and ultimately converging onto microglia within the fetal brain, resulting in the long-term alteration of brain function and behavior.

## Conclusions

We observed that maternal psychological stress induced by resource deprivation during late pregnancy increased the vulnerability of murine offspring, particularly males, to *in utero* air pollutant exposure. Furthermore, developmental exposure to maternal stress and air pollution, similar to other environmental compounds such as pesticides and lipopolysaccharide (Ling et al. 2004), may have a long-lasting impact on microglial function and neuroinflammation. Future studies aimed at elucidating the complex interactions of psychosocial and chemical stressors will be critical for informing environmental and public health policy and identifying effective interventions.

## Supplemental Material

(389 KB) PDF

## References

[r1] Adler NE, Rehkopf DH (2008). U.S. disparities in health: descriptions, causes, and mechanisms.. Annu Rev Public Health.

[r2] Arbour NC, Lorenz E, Schutte BC, Zabner J, Kline JN, Jones M (2000). *TLR4* mutations are associated with endotoxin hyporesponsiveness in humans. Nat Genet.

[r3] Auten RL, Gilmour MI, Krantz QT, Potts EN, Mason SN, Foster WM (2012). Maternal diesel inhalation increases airway hyperreactivity in ozone-exposed offspring.. Am J Respir Cell Mol Biol.

[r4] Auten RL, Potts EN, Mason SN, Fischer B, Huang Y, Foster WM (2009). Maternal exposure to particulate matter increases postnatal ozone-induced airway hyperreactivity in juvenile mice.. Am J Respir Crit Care Med.

[r5] Avishai-Eliner S, Eghbal-Ahmadi M, Tabachnik E, Brunson KL, Baram TZ (2001). Down-regulation of hypothalamic corticotropin-releasing hormone messenger ribonucleic acid (mRNA) precedes early-life experience-induced changes in hippocampal glucocorticoid receptor mRNA.. Endocrinology.

[r6] Bauernfeind FG, Horvath G, Stutz A, Alnemri ES, MacDonald K, Speert D (2009). Cutting edge: NF-κB activating pattern recognition and cytokine receptors license NLRP3 inflammasome activation by regulating NLRP3 expression.. J Immunol.

[r7] Bianchi ME (2007). DAMPs, PAMPs and alarmins: all we need to know about danger.. J Leukoc Biol.

[r8] Bilbo SD, Newsum NJ, Sprunger DB, Watkins LR, Rudy JW, Maier SF (2007). Differential effects of neonatal handling on early life infection-induced alterations in cognition in adulthood.. Brain Behav Immun.

[r9] Bilbo SD Schwarz JM 2009 Early-life programming of later-life brain and behavior: a critical role for the immune system. Front Behav Neurosci 3 14; 10.3389/neuro.08.014.2009 19738918 PMC2737431

[r10] Black RA, Kronheim SR, Cantrell M, Deeley MC, March CJ, Prickett KS (1988). Generation of biologically active interleukin-1β by proteolytic cleavage of the inactive precursor.. J Biol Chem.

[r11] Bolton JL, Smith SH, Huff NC, Gilmour MI, Foster WM, Auten RL (2012). Prenatal air pollution exposure induces neuroinflammation and predisposes offspring to weight gain in adulthood in a sex-specific manner.. FASEB J.

[r12] Boulanger LM (2009). Immune proteins in brain development and synaptic plasticity.. Neuron.

[r13] Caldji C, Tannenbaum B, Sharma S, Francis D, Plotsky PM, Meaney MJ (1998). Maternal care during infancy regulates the development of neural systems mediating the expression of fearfulness in the rat.. Proc Natl Acad Sci USA.

[r14] Cao X Laplante DP Brunet A Ciampi A King S 2012 Prenatal maternal stress affects motor function in 5½-year-old children: Project Ice Storm. Dev Psychobiol; 10.1002/dev.21085 [Online 9 November 2012] 23143986

[r15] Case A, Fertig A, Paxson C (2005). The lasting impact of childhood health and circumstance.. J Health Econ.

[r16] Caso JR, Pradillo JM, Hurtado O, Leza JC, Moro MA, Lizasoain I (2008). Toll-like receptor 4 is involved in subacute stress–induced neuroinflammation and in the worsening of experimental stroke.. Stroke.

[r17] Castagné V, Moser P, Roux S, Porsolt RD. 2010 Rodent models of depression: Forced swim and tail suspension behavioral despair tests in rats and mice Curr Protoc Pharmacol Chapter 5:Unit 5.8; 10.1002/0471141755.ph0508s49 22294373

[r18] Clougherty JE Levy JI Kubzansky LD Ryan PB Suglia SF Canner MJ 2007 Synergistic effects of traffic-related air pollution and exposure to violence on urban asthma etiology. Environ Health Perspect 115 8 1140 1146; 10.1289/ehp.9863 17687439 PMC1940095

[r19] Dantzer R, O’Connor JC, Freund GG, Johnson RW, Kelley KW (2008). From inflammation to sickness and depression: when the immune system subjugates the brain.. Nat Rev Neurosci.

[r20] De Pablos R, Villaran R, Argüelles S, Herrera A, Venero J, Ayala A (2006). Stress increases vulnerability to inflammation in the rat prefrontal cortex.. J Neurosci.

[r21] de Wit M Wiaterek GK Gray ND Goulet KE Best AM Clore JN 2010 Relationship between alcohol use disorders, cortisol concentrations, and cytokine levels in patients with sepsis. Crit Care 14 6 R230; 10.1186/cc9385 21176217 PMC3219986

[r22] Deverman BE, Patterson PH (2009). Cytokines and CNS development.. Neuron.

[r23] Evans GW, Kantrowitz E (2002). Socioeconomic status and health: the potential role of environmental risk exposure.. Annu Rev Public Health.

[r24] Fang F Höglund CO Arck P Lundholm C Långström N Lichtenstein P 2011 Maternal bereavement and childhood asthma—analyses in two large samples of Swedish children. PLoS One 6 11 e27202; 10.1371/journal.pone.0027202 22087265 PMC3210147

[r25] Flannery KA, Liederman J, Daly L, Schultz J (2000). Male prevalence for reading disability is found in a large sample of black and white children free from ascertainment bias.. J Int Neuropsychol Soc.

[r26] Foster WM, Walters DM, Longphre M, Macri K, Miller LM (2001). Methodology for the measurement of mucociliary function in the mouse by scintigraphy.. J Appl Physiol.

[r27] Frank MG, Baratta MV, Sprunger DB, Watkins LR, Maier SF (2007). Microglia serve as a neuroimmune substrate for stress-induced potentiation of CNS pro-inflammatory cytokine responses.. Brain Behav Immun.

[r28] Frank MG, Miguel ZD, Watkins LR, Maier SF (2010). Prior exposure to glucocorticoids sensitizes the neuroinflammatory and peripheral inflammatory responses to *E. coli* lipopolysaccharide.. Brain Behav Immun.

[r29] Frank MG, Watkins LR, Maier SF (2011). Stress-and glucocorticoid-induced priming of neuroinflammatory responses: potential mechanisms of stress-induced vulnerability to drugs of abuse.. Brain Behav Immun.

[r30] Garate I, Garcia-Bueno B, Madrigal JL, Caso JR, Alou L, Gomez-Lus ML (2013). Stress-induced neuroinflammation: role of the toll-like receptor-4 pathway.. Biol Psychiatry.

[r31] Ginhoux F, Greter M, Leboeuf M, Nandi S, See P, Gokhan S (2010). Fate mapping analysis reveals that adult microglia derive from primitive macrophages.. Science.

[r32] Hackman DA, Farah MJ, Meaney MJ (2010). Socioeconomic status and the brain: mechanistic insights from human and animal research.. Nat Rev Neurosci.

[r33] Hsiao EY, Patterson PH (2012). Placental regulation of maternal-fetal interactions and brain development.. Dev Neurobiol.

[r34] Kouduka M Matsuoka A Nishigaki K 2006 Acquisition of genome information from single-celled unculturable organisms (radiolaria) by exploiting genome profiling (GP). BMC Genomics 7 1 135; 10.1186/1471-2164-7-135 16740170 PMC1523345

[r35] Latz E (2010). The inflammasomes: mechanisms of activation and function.. Curr Opin Immunol.

[r36] Levesque S Taetzsch T Lull ME Kodavanti U Stadler K Wagner A 2011 Diesel exhaust activates and primes microglia: air pollution, neuroinflammation, and regulation of dopaminergic neurotoxicity. Environ Health Perspect 119 1149 1155; 10.1289/ehp.1002986 21561831 PMC3237351

[r37] Ling EA, Wong WC (2004). The origin and nature of ramified and amoeboid microglia: a historical review and current concepts.. Glia.

[r38] Ling Z, Chang QA, Tong CW, Leurgans SE, Lipton JW, Carvey PM (2004). Rotenone potentiates dopamine neuron loss in animals exposed to lipopolysaccharide prenatally.. Exp Neurol.

[r39] Myers MM, Brunelli SA, Squire JM, Shindeldecker RD, Hofer MA (1989). Maternal behavior of SHR rats and its relationship to offspring blood pressures.. Dev Psychobiol.

[r40] Needleman HL, Schell A, Bellinger D, Leviton A, Allred EN (1990). The long-term effects of exposure to low doses of lead in childhood.. N Engl J Med.

[r41] Phillips R, LeDoux J (1992). Differential contribution of amygdala and hippocampus to cued and contextual fear conditioning.. Behav Neurosci.

[r42] Reijneveld SA, Schene AH (1998). Higher prevalence of mental disorders in socioeconomically deprived urban areas in the netherlands: community or personal disadvantage?. J Epidemiol Community Health.

[r43] Rice CJ, Sandman CA, Lenjavi MR, Baram TZ (2008). A novel mouse model for acute and long-lasting consequences of early life stress.. Endocrinology.

[r44] Schwarz JM, Bilbo SD (2013). Adolescent morphine exposure affects long-term microglial function and later-life relapse liability in a model of addiction.. J Neurosci.

[r45] Schwarz JM, Hutchinson MR, Bilbo SD (2011). Early-life experience decreases drug-induced reinstatement of morphine CPP in adulthood via microglial-specific epigenetic programming of anti-inflammatory IL-10 expression.. J Neurosci.

[r46] Seguin L, Potvin L, St-Denis M, Loiselle J (1995). Chronic stressors, social support, and depression during pregnancy.. Obstet Gynecol.

[r47] Shankardass K, McConnell R, Jerrett M, Milam J, Richardson J, Berhane K (2009). Parental stress increases the effect of traffic-related air pollution on childhood asthma incidence.. Proc Natl Acad Sci USA.

[r48] Shepherd JK, Grewal SS, Fletcher A, Bill DJ, Dourish CT (1994). Behavioural and pharmacological characterisation of the elevated “zero-maze” as an animal model of anxiety.. Psychopharmacology.

[r49] Stone JL, Merriman B, Cantor RM, Yonan AL, Gilliam TC, Geschwind DH (2004). Evidence for sex-specific risk alleles in autism spectrum disorder.. Am J Hum Genet.

[r50] Susser EB, Brown A, Matte TD (1999). Prenatal factors and adult mental and physical health.. Can J Psychiatry.

[r51] Thompson AL, Johnson BT, Sempowski GD, Gunn MD, Hou B, DeFranco AL (2012). Maximal adjuvant activity of nasally delivered IL-1α requires adjuvant-responsive CD11c^+^ cells and does not correlate with adjuvant-induced in vivo cytokine production.. J Immunol.

[r52] Volk HE, Lurmann F, Penfold B, Hertz-Picciotto I, McConnell R (2013). Traffic-related air pollution, particulate matter, and autism.. JAMA Psychiatry.

[r53] Weiss B, Landrigan PJ (2000). The developing brain and the environment: an introduction.. Environ Health Perspect.

[r54] Williamson LL, Sholar PW, Mistry RS, Smith SH, Bilbo SD (2011). Microglia and memory: modulation by early-life infection.. J Neurosci.

[r55] Wright RJ (2009). Moving towards making social toxins mainstream in children’s environmental health.. Curr Opin Pediatr.

[r56] Yirmiya R, Goshen I (2011). Immune modulation of learning, memory, neural plasticity and neurogenesis.. Brain Behav Immun.

