# Supplementary material for: Maternal Stress and Effects of Prenatal Air Pollution on Offspring Mental Health Outcomes in Mice
Source: Environ Health Perspect. 2013 Jul 3;121(9):1075–82. doi: 10.1289/ehp.1306560 (PMC3764088; doi:10.1289/ehp.1306560)
Supplement: (389 KB) PDF [file ehp.1306560.s001.pdf]

SUPPLEMENTAL MATERIAL

Maternal Stress and Effects of Prenatal Air Pollution on Offspring Mental Health Outcomes in Mice

Jessica L. Bolton[1*], Nicole C. Huff[1], Susan H. Smith[1], S. Nicholas Mason[2], W. Michael Foster[3], Richard L. Auten[2], and Staci D. Bilbo[1]

Table of Contents

Page 2-6: Supplemental Methods

Page 7: Table S1

Page 8: Table S2

Page 9: Figure S1

Page 10: Figure S2

Page 11: References

SUPPLEMENTAL METHODS

Animals

We housed adult male and female C57BL/6 mice in individually ventilated, microisolator polypropylene cages with specialized bedding (AlphaDri; to avoid exposure to potentially confounding antigens that can be found in typical bedding) and *ad libitum* food (PicoLab Mouse Diet 5058, Lab-Diet, Philadelphia, PA) and filtered water. The colony was maintained at 22°C on a 12:12-hr light-dark cycle (lights off at 9 AM). Following acclimation to laboratory conditions for 1 week, we placed males with 2 females each for breeding, for a maximum of 2 weeks. We examined females twice daily for evidence of a vaginal plug [confirmation of successful mating, considered to be embryonic day (E)0], at which point we separated them from the male and paired them with another successfully mated female. Females were bred in four separate cohorts (n=46 total dams), and each cohort was used for a separate analysis: 1) fetal brain cytokine protein, 2) P30 brain gene expression, 3) adult behavior and brain cytokine protein, and 4) adult microglial isolations and gene expression. In addition, one pup was randomly selected at P1 from litters across the second and third cohorts for CORT analysis.

DEP Exposures

Diesel exhaust was generated using a 4.8kW direct injection single-cylinder 320 mL displacement Yanmar L70V diesel generator operating at a constant 3.500 rpm. Resistance heating elements were used to impose a constant 3kW load to the engine. Low sulfur diesel fuel was used. Diesel exhaust particles were collected using an electrostatic precipitator. Chemical analysis of particles yielded an organic carbon/elemental ratio = 0.63, with extractable organic

matter = 39.8%. The particle size distribution ranged from 18-200 μm [further detailed analyses may be found in (Auten et al. 2012)].

We prepared 1 mg/ml DEP suspensions by briefly sonicating in phosphate buffered saline pH 7.2 0.05% Tween-20, then administered 50 μl DEP in vehicle or vehicle alone to dams by oropharyngeal aspiration under 2% isoflurane anesthesia. Following brief anesthetization, we suspended mice by frontal incisors, and upon the beginning of recovery with the onset of deeper inspirations, we gave the dose by holding the tongue with forceps and administering the suspension with a micropipettor to the posterior oropharynx. Bronchoalveolar lavage two days after administration demonstrates DEP in alveolar macrophages, and pilot studies show distal alveolar deposition at necropsy (data not shown). Furthermore, gross inspection of placentae rarely reveals any abnormalities, besides the occasional fetal resorption, and placental weights are not affected by maternal treatment (data not shown).

Fetal and Adult Brain Cytokine Analysis

We measured protein levels of IL-1β and IL-10 in fetal and adult brain as described previously (Williamson et al. 2011), except for minor changes. Previous studies in our lab (unpublished data) have shown that the protein measurement works best when myelin is removed via lipid extraction (Hara and Radin 1978) of the brain homogenates prior to analysis. Thus, we added 1 ml of hexanes (~2 × volume of homogenate; Mallinckrodt Baker, Inc., Philipsburg, NJ) to each sample following tissue homogenization and sonication. We vortexed and centrifuged samples briefly at 13,000 × *g* and then extracted the aqueous layer. Finally, we removed residual hexanes by decantation after freezing the aqueous fractions on dry ice for ~3 min.

P30 Neuroimmune Gene Expression

We isolated total RNA from P30 half-brains using TRIzol (Chomczynski and Sacchi 1987). Subsequently, we synthesized cDNA from 100 ng of RNA using the QuantiTect reverse transcription kit (Qiagen, Inc., Valencia, CA) and a 15 min cDNA synthesis reaction. We performed quantitative real-time PCR (qRT-PCR) using a QuantiFast SYBR Green PCR kit (Qiagen) on a Mastercycler ep *realplex* (Eppendorf, Hauppauge, NY). For each reaction, we added 1 µl of cDNA to 12 µl of master mix containing specific primers for GAPDH, TLR4, or caspase-1 (Qiagen). For analysis, we determined the threshold cycle (C_T) for each reaction and calculated relative gene expression using the $2^{-\Delta\Delta Ct}$ method (Livak and Schmittgen 2001; Pfaffl 2001; Williamson et al. 2011).

Behavioral Procedures

Maternal behavior assessment

We observed dams with their litters postnatal day (P)2-P9 twice daily, once each during the light (8-9 AM) and dark (4-5 PM) cycles. During each session, we scored dams (n=3-7/group) for 'on nest', nursing, and licking and grooming (LG) behavior every 5 min for 1 hr (Myers et al. 1989). As previously described, the frequency of occurrence for some of the behaviors (particularly LG) was rare, so we summed data for each behavior across all 16 observation sessions and calculated a percent of time spent performing each behavior for each dam. We took care not to disturb dams during observation sessions, and changed their cages only upon completion of the final observation on P9.

Contextual and auditory cue fear conditioning

Apparatus. The conditioning context consisted of chambers (20 cm × 19 cm × 33 cm) made of clear Plexiglas with metal side walls (Coulborn Instruments, Whitehall, PA), placed inside a black Plexiglas box (41.9 cm × 41.9 cm × 45.4 cm) with an open front to allow viewing of the animals, but to prevent them from seeing each other. Each chamber has a ceiling-mounted speaker capable of emitting a phasic auditory cue (2976-Hz tone presented at 76 dB) and a white house light. A removable floor of stainless steel rods (0.5 cm diameter, spaced 1.75 cm apart) is wired to a shock generator and scrambler. The chambers were cleaned with 70% ethanol followed by water before each animal.

Procedure. We allowed mice to explore the chamber for 2 min before the onset of a 15 sec tone, followed immediately by a 2 sec footshock (0.8 mA). The same tone-shock pairing was repeated 90 and 180 sec later, and immediately after the termination of the third footshock, we removed mice from the chamber. We tested mice 48 hr later for contextual fear memory, as assessed by freezing behavior, in the original conditioning chamber, but in the absence of auditory cues or footshocks. Freezing represents the dominant defensive fear response of rodents and is characterized by an immediate suppression of behavior and immobility (except for respiration) (Blanchard et al. 1976). We began scoring 10 sec after placing the animal into the chamber, and continued every 10 sec for the remainder of the 6-min test. For assessment of auditory-cue fear memory (3 hr after contextual fear test), testing occurred in a novel triangular chamber (31.8 cm × 26.7 cm × 38.1 cm × 25.4 cm) with clear Plexiglas walls and floor, illuminated by a 7-W clear, red light bulb. We again assessed freezing for 6 min. The auditory conditioned stimulus (CS) was absent for the first 3 min (pre-CS period) and present for the last 3 min (CS period). Freezing during the pre-CS period indicates generalized fear of a novel environment, whereas

freezing during the CS period indicates fear specific to the auditory cue (Frankland et al. 1998; Paylor et al. 1994).

Elevated zero-maze

The maze has an elevated (49.5 cm high) circular lane (4.5 cm wide) divided into four quadrants. Two opposite quadrants are enclosed by walls (15.9 cm high), whereas the remaining two quadrants are left exposed. We placed each mouse onto the center of an open arm, and scored the total time they spent in the closed arms out of 5 min. An increase in time spent in the closed arms is indicative of increased anxiety (Shepherd et al. 1994). We cleaned the maze between animals.

Forced swim test

The apparatus is a Plexiglas cylinder (20.3 cm diameter × 49.5 cm high) filled 2/3-full with 22°C water. We lowered each mouse into the center of the water-filled cylinder, and then scored them for their duration of immobility for 6 min. Immobility was defined as the absence of all swimming, except for the subtle motions required to keep the head above water (Castagné et al. 2010).

Gene Expression of Isolated CD11b+ and CD11b- Cells

We isolated total RNA from CD11b+ and CD11b- cells using TRIzol (Chomczynski and Sacchi 1987), adding glycogen (Invitrogen, Grand Island, New York) to the aqueous phase to maximize RNA precipitation. Next, we synthesized cDNA from 100 ng of RNA as above, but using a 30 min cDNA synthesis reaction, followed by qRT-PCR as above. We purchased primers for CD11b, caspase-1, IL-1β, BDNF, and GFAP from Qiagen and designed primer pairs for 18S, IL-10, and TLR4 (see Table S1) as previously described (Williamson et al. 2011).

Table S1. We obtained PCR primers from Sigma-Aldrich. We subsequently determined optimal annealing temperatures for each primer pair by running a temperature gradient, and verified specificity by melt-curve analysis.

Gene	Forward Primer (5'-3')	Reverse Primer (5'-3')
Sry	TGGGCTGGACTAGGGAGGTCC	TGCTGGGCCAACTTGTGCCT
18S	GAATAATGGAATAGGACCGC	CTTTCGCTCTGGTCCGTCTT
Il10	GGACAACATACTGCTAACCGAC	TGCTCCTTGATTTCTGGGC
Tlr4	GCTGGATTTATCCAGGTGTG	AGTCCAGAGAAACTTCCTGG

Table S2. Litter sizes and sex ratios by maternal treatment. All values are mean ± SEM.

Treatment	Litter Size	Sex Ratio (% Males)
VEH/Control	9.0 ± 0.5	56.9% ± 6.5%
DEP/Control	8.7 ± 0.4	57.5% ± 7.3%
VEH/NR	8.5 ± 0.8	59.8% ± 3.1%
DEP/NR	8.3 ± 0.5	53.6% ± 5.8%

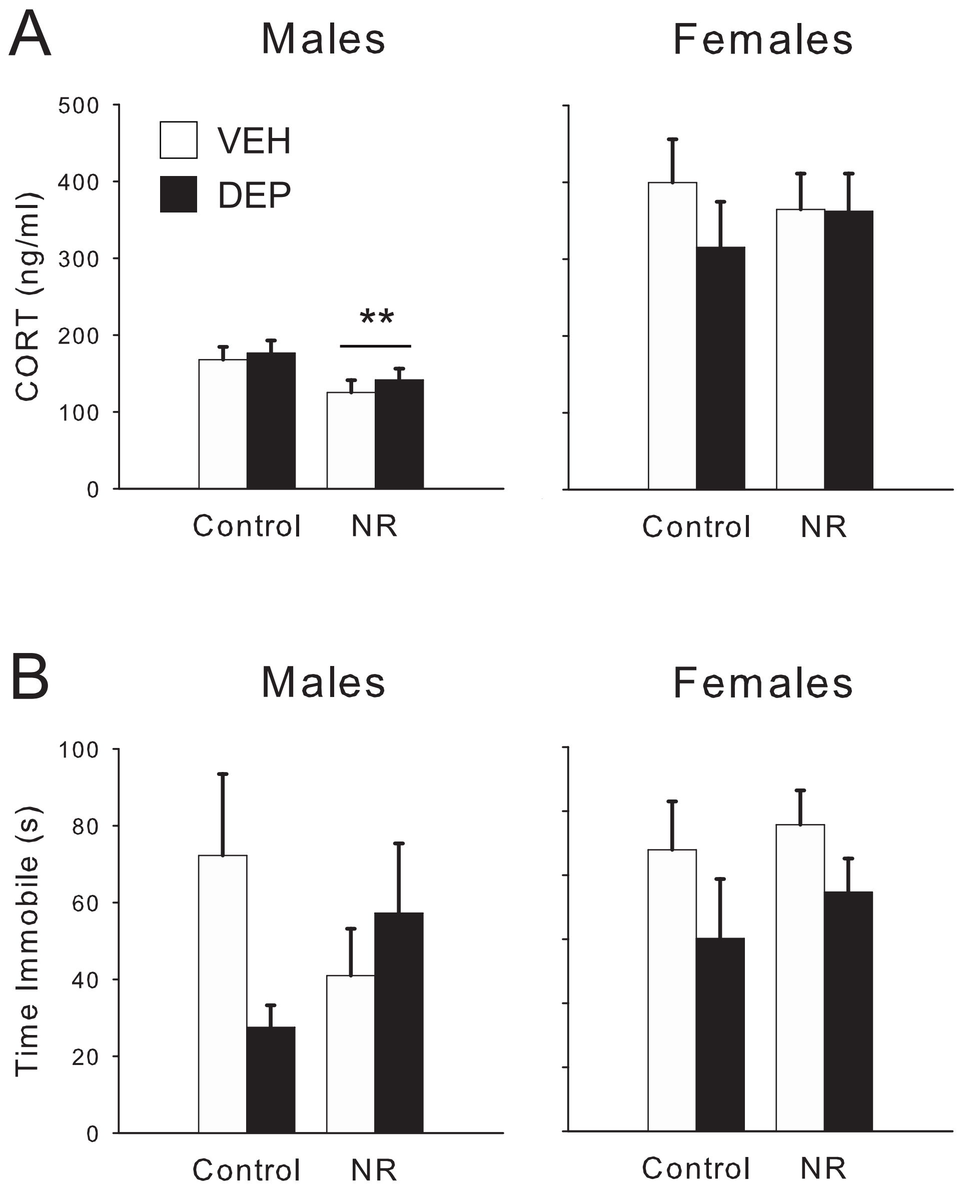

Figure S1. (*A*) Male offspring exposed to prenatal NR exhibited a decreased level of serum CORT immediately following the elevated zero maze test, whereas there were no differences in female offspring. (*B*) Neither males nor females displayed any significant changes in depressive-like behavior in the forced swim test. Data are mean of n=7-9/group ± SEM (***p*<0.05, NR vs. Control).

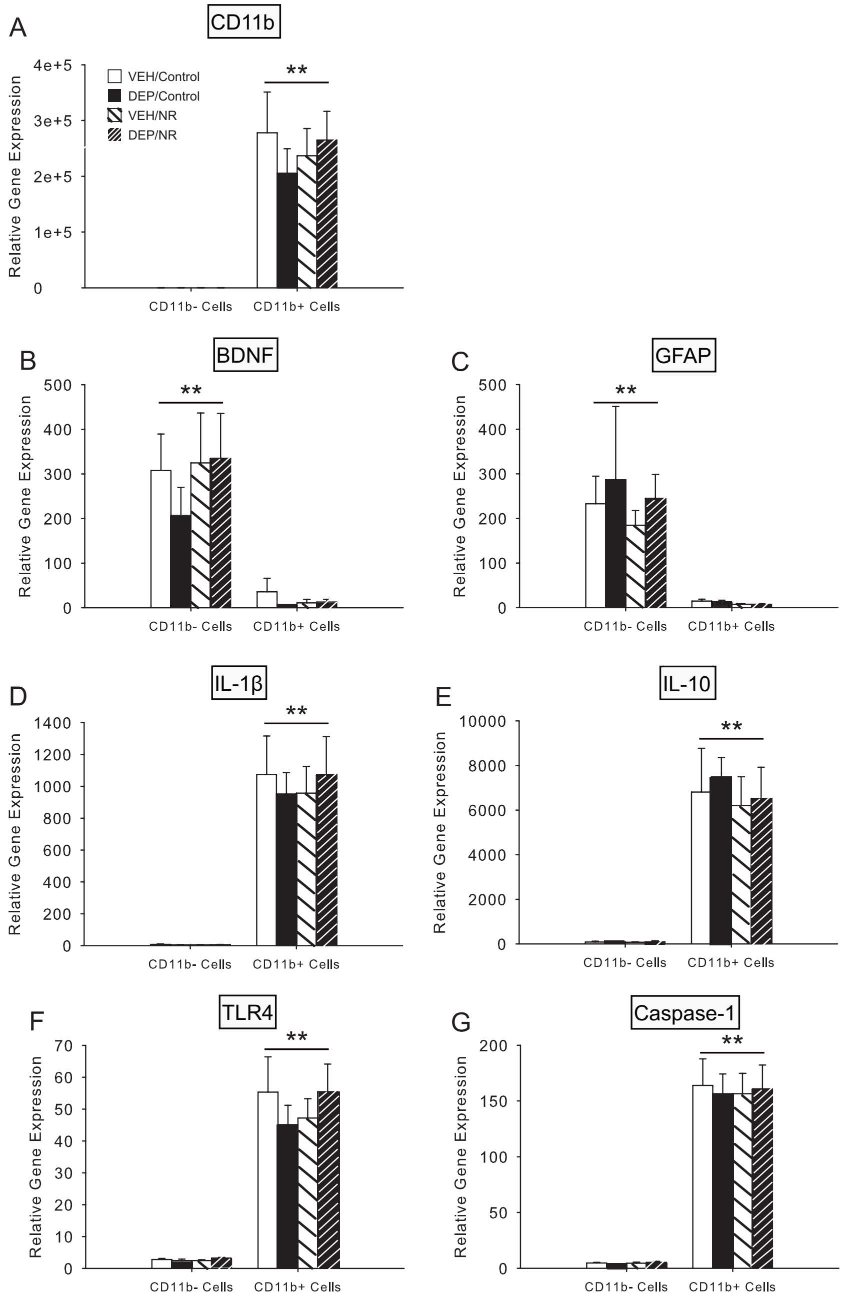

Figure S2. Isolated CD11b+ cells (microglia) expressed markedly higher levels of CD11b (*A*), IL-1β (*D*), IL-10 (*E*), TLR4 (*F*), and caspase-1 (*G*) than did CD11b- cells (neurons, astrocytes). CD11b- cells expressed significantly higher levels of BDNF (*B*) and GFAP (*C*). Data are mean of n=5/group ± SEM (***p*<0.05, CD11b+ vs. CD11b- cells).

References

Auten RL, Gilmour MI, Krantz QT, Potts EN, Mason SN, Foster WM. 2012. Maternal diesel inhalation increases airway hyperreactivity in ozone-exposed offspring. Am J Respir Cell Mol Biol 46(4):454-460.

Blanchard RJ, Fukunaga KK, Blanchard DC. 1976. Environmental control of defensive reactions to footshock. Bull Psychon Soc 8(129):40.

Castagné V, Moser P, Roux S, Porsolt RD. 2010. Rodent models of depression: Forced swim and tail suspension behavioral despair tests in rats and mice. Curr Protoc Pharmacol:5.8.1-5.8.14.

Chomczynski P, Sacchi N. 1987. Single-step RNA isolation from cultured cells or tissues. Anal Biochem 162:156-259.

Frankland PW, Cestari V, Filipkowski RK, McDonald RJ, Silva AJ. 1998. The dorsal hippocampus is essential for context discrimination but not for contextual conditioning. Behav Neurosci 112(4):863.

Hara A, Radin NS. 1978. Lipid extraction of tissues with a low-toxicity solvent. Anal Biochem 90(1):420-426.

Livak KJ, Schmittgen TD. 2001. Analysis of relative gene expression data using real-time quantitative PCR and the $2^{-\Delta\Delta CT}$ method. Methods 25(4):402-408.

Myers MM, Brunelli SA, Squire JM, Shindeldecker RD, Hofer MA. 1989. Maternal behavior of SHR rats and its relationship to offspring blood pressures. Dev Psychobiol 22(1):29-53.

Paylor R, Tracy R, Wehner J, Rudy JW. 1994. DBA/2 and C57BL/6 mice differ in contextual fear but not auditory fear conditioning. Behav Neurosci 108(4):810.

Pfaffl MW. 2001. A new mathematical model for relative quantification in real-time RT–PCR. Nucleic Acids Res 29(9):e45-e45.

Shepherd JK, Grewal SS, Fletcher A, Bill DJ, Dourish CT. 1994. Behavioural and pharmacological characterisation of the elevated "zero-maze" as an animal model of anxiety. Psychopharmacology 116(1):56-64.

Williamson LL, Sholar PW, Mistry RS, Smith SH, Bilbo SD. 2011. Microglia and memory: Modulation by early-life infection. J Neurosci 31(43):15511-15521.
